# An Environmental Risk Assessment for Human-Use Trimethoprim in European Surface Waters

**DOI:** 10.3390/antibiotics2010115

**Published:** 2013-03-18

**Authors:** Jürg Oliver Straub

**Affiliations:** F. Hoffmann-La Roche Ltd., Group SHE, CH-4070 Basle, Switzerland; E-Mail: juerg.straub@roche.com; Tel.: +41-616-885-781; Fax: +41-616-881-920

**Keywords:** trimethoprim, environmental exposure, environmental effects, environmental risk assessment, surface waters, Europe

## Abstract

An environmental risk assessment (ERA) for the aquatic compartment in Europe from human use was developed for the old antibiotic Trimethoprim (TMP), comparing exposure and effects. The exposure assessment is based on European risk assessment default values on one hand and is refined with documented human use figures in Western Europe from IMS Health and measured removal in wastewater treatment on the other. The resulting predicted environmental concentrations (PECs) are compared with measured environmental concentrations (MECs) from Europe, based on a large dataset incorporating more than 1800 single MECs. On the effects side, available chronic ecotoxicity data from the literature were complemented by additional, new chronic results for fish and other organisms. Based on these data, chronic-based deterministic predicted no effect concentrations (PNECs) were derived as well as two different probabilistic PNEC ranges. The ERA compares surface water PECs and MECs with aquatic PNECs for TMP. Based on all the risk characterization ratios (PEC÷PNEC as well as MEC÷PNEC) and risk graphs, there is no significant risk to surface waters.

## 1. Introduction

The topic of pharmaceuticals in the environment (PIE) has gained a lot of attention in environmental discussions. Active pharmaceutical ingredients (APIs) are suspected of causing unintended adverse effects in environmental compartments, based on their intended property of high biological activity. For human APIs, which are excreted into wastewater, this primarily means concern for the sewage treatment plants (STPs) or surface waters. Such concerns have been fuelled by ubiquitous detections of APIs in STP effluents and surface waters since the 1970s, in concentrations in the ng/L to µg/L range. It is mostly older APIs that are regularly monitored and detected. While for the registration of new APIs an environmental risk assessment (ERA) has been requested in the European Union since the early 1990s [1], this was not the case beforehand, meaning that exactly for these older APIs there often is a lack of environmental fate and toxicity data.

The old antibiotic trimethoprim (TMP) was first put on the market by F. Hoffmann-La Roche Ltd (Roche) in the 1960s in combination with sulfamethoxazole (SMX) under the brand name of Bactrim^®^. TMP has been regularly detected in the environment. Like all antibiotics, TMP has come under suspicion for the potential of selecting for, maintaining or increasing antibiotic resistance in environmental bacteria. The first in-depth aquatic ERA for TMP is presented here. It is based on both predicted and measured environmental concentrations (PECs and MECs, respectively) and on published and new chronic ecotoxicity data. Some of the latter were specifically commissioned in order to produce a solid effects assessment for TMP. Acute ecotoxicity data are integrated as well. In view of sufficient data available, this ERA was supplemented with a probabilistic comparison of percent-ranked MECs and chronic effects species sensitivity distributions in addition to the standard deterministic procedures.

## 2. Results and Discussion

### 2.1. Trimethoprim Pharmacological Data

#### 2.1.1. TMP Mode of Action

The diaminopyrimidine TMP (2,4-diamino-5-(3,4,5-trimethoxybenzyl)pyrimidine; CAS Number 738-70-5) [2] is a bacteriostatic API that interferes with the bacterial dihydrofolate reductase enzyme, inhibiting the synthesis of tetrahydrofolic acid [2]. Bacteria are unable to take up folic acid from the environment, including their infection host in case of pathogenic species, and are dependent on their own *de novo* synthesis. Inhibition of dihydrofolate reductase starves the bacteria of nucleotides necessary for DNA replication. TMP is generally used in combination with sulfonamide antibiotics (mainly SMX), which interfere with another step of bacterial folate synthesis pathway; in combination, TMP and SMX act synergistically. 

#### 2.1.2. TMP Adsorption, Metabolism and Excretion

TMP is rapidly absorbed after oral administration and widely distributed around the body to tissues and fluids. Serum therapeutic concentrations range from 1.5–2.5 mg/L up to 9 mg/L [3]. Metabolic reactions include oxidation of the methylene group to a hydroxymethyl group, *N*-oxidation, *O*-de-methylation and hydroxylation in phase-1 metabolism as well as conjugation with glucuronic acid or sulfate in phase 2. Around 10%–20% of a dose is metabolized. The metabolites are excreted in the urine as conjugates, but the greater part of the dose is excreted as unchanged drug. Urinary excretion is *p*H-dependent and is increased in acidic urine. About 40%–75% of a dose is excreted in 24 h, up to 60% being in the form of unchanged drug, with about 4% each as the 3'-hydroxymethyl and 4'-hydroxymethyl metabolites and 2% as the N1-oxide. Less than 4% is eliminated in the faeces. The plasma half-life ranges from 8 to 17 h with an average of 11 h [2,3]. The World Health Organization defined daily dose of TMP is 400 mg [4]. This value will later be used for the first PEC derivation.

#### 2.1.3. TMP Toxicity

TMP is not particularly toxic to humans and mammals by oral administration in the short or longer term, however, it can be irritant and sensitizing [2]. It was mutagenic in a bacterial test system and at high doses it can be teratogenic and embryotoxic through its mode of action, folate antagonism [2], as folic acid is required for normal development. However, due to these mutagenic and reprotoxic properties, TMP is classified by default as T for toxic for a persistence, bioaccumulation and toxicity (PBT) assessment.

### 2.2. TMP Environmental Fate and Concentrations

The basic data for the environmental fate and effects of TMP are listed in tables in the Appendix of this publication, starting on Page 136, for better readability of the text. A discussion of the most important, selected values from these tables is presented in the following sections.

#### 2.2.1. Physico-Chemical Data for TMP

Physico-chemical data for TMP are listed in the Appendix in Table A1, Page 136 *ff* [2,3,5,6,7,8,9,10,11,12,13,14,15,16,17,18,19,20,21,22,23,24,25,26,27]. TMP is an organic base with a reasonably high water solubility of ~300 mg/L and a first base dissociation constant *p*K_a_ around the neutral *p*H point [2]. There are no hydrolysable bonds. The melting point is around 200 °C [2], vapor pressure is low at ~1.32 × 10^–6^ Pa [5], hence the Henry’s Law Constant is low as well and the substance will not volatilize from water. In addition, with a first base *p*K_a_ around 7 [2,3,9], TMP is at least partly dissociated in most environmental waters, *i.e.*, it will be more hydrophilic and will volatilize even less. With an *n*-octanol/water partition coefficient logKow between 0.64 and 1.115 [2,12] TMP is not particularly lipophilic. Therefore, neither strong adsorption to organic substrates nor bioaccumulation would be expected. In confirmation, moderate to low adsorption constants to organic carbon (OC), activated sludge (AS) and soil have been published [13,14,15,16,17,18,19,20,21,22,23,24,25,26,27], although Lin & Gan [16] noted strong adsorption in one soil beside moderate adsorption in others. Specifically, sorption to AS in sewage treatment plants (STPs) has been independently described as ‘negligible’ [25,26,27]. However, sorption should still be kept in mind as Trapp *et al.* [28] have shown using physicochemical activity-based environmental fate modeling that as a weak base, TMP is non-dissociated and thus more prone to sorption or bioaccumulation at a higher environmental *p*H of 9 than at *p*H 6 where TMP is mostly dissociated. In general, based on this low to moderate sorption, most TMP is expected to remain predominantly in the aqueous phase, meaning that little is removed to sludge in STPs, the exposure of soil by landspreading of digested surplus sludge is low, mobility in soils is high and little will partition from surface waters to sediment. 

#### 2.2.2. Biodegradation, Environmental Fate and Bioaccumulation Data for TMP

The available literature data for TMP for biodegradation, removal in STPs, environmental fate and derived half-lives as well as bioaccumulation are collated in Table A2, Table A3, Table A4, Table A5 at the end (Page 138 *ff*).

##### 2.2.2.1. Biodegradability of TMP (Table A2) [18,19,21,25,26,29,30,31,32,33,34,35]

TMP is recalcitrant to biodegradation in standard ready and inherent tests [18,21,29,31] and also in a standard STP model test at low concentration [32]. This first impression may be misleading, however, as on one hand, significant cometabolic degradation was observed in a closed bottle test with sodium acetate in the toxicity control [29]. Moreover, good removal (>50%) was seen in those tests performed with aerobic AS with a long sludge retention time (SRT), *i.e.*, a high sludge age [21,25,33,34,35]. Indeed, as consistently shown by Göbel *et al.* [19,26], Perez *et al.* [34] and Schröder *et al.* [35], who compared the removal in different steps of STPs, low removal was found in inocula with a short SRT, e.g., from primary sludge or young AS, but high removal was noted for inocula with a high SRT, *i.e.*, nitrifying AS and sand filters. Similarly, rapid primary degradation of TMP was also shown by Löffler & Ternes [36] for natural sediments and by Schmidt *et al.* [37] during river bank filtration. In addition, Bundschuh *et al.* [30] determined that TMP is even rapidly degraded in a ready-type system, exposing fallen leaves in natural water to low concentrations of TMP, where they determined ~80% degradation in 7 days. A similar difference may also exist for anaerobic degradation as Gartiser *et al.* [21] recorded no significant methane production in a standard ISO 11734 anaerobic degradation test, while other investigations with surplus sludge from an anaerobic digestor [19], with manure and anaerobic bacteria [38] or in pig slurry [39] found high and rapid removal. In soil [13,40] and seawater [41], however, biodegradation seems to be slow with correspondingly long half-lives of around or more than 100 days. 

##### 2.2.2.2. Removal of TMP during Sewage Treatment (Table A3) [19,23,25,36,37,38,42,43,44,45,46,47,48,49,50,51,52,53,54,55,56,57,58,59,60,61,62,63,64,65,66,67,68,69,70,71,72,73,74]

The above differences, mainly relating to SRT respectively nitrifying conditions, are probably responsible for the inordinately high range of removal noted for many different STPs in Europe, North America and the Far East. These include negative removal, which may signify cleavage of glucuronide or sulfate conjugates [75], and range up to nearly 100% [19,23,48,52,53,54,55,56,57,58,59,60,61,62,63]. Some of the negative removal rates, like the extreme value of −550% described by Lindberg *et al.* [45] for one STP in Sweden, are highly improbable, seeing as 60%–80% of ingested TMP is excreted as the parent and only 20%–40% as metabolites and conjugates [2,3]. Therefore, conjugate cleavage of 550% is quite impossible, but either sampling, synchronization or analytical problems are suspected. In conclusion, for TMP in STPs there is but minor removal during inadequate primary and secondary treatment [19,34], but nitrifying sludge is able to biodegrade TMP [25,34], suggesting an important role for both aerobic conditions [76] and in particular for long SRTs in secondary treatment [55,62]. Similarly, anaerobic degradation may range from low [21] to rather high [19,38,39]. 

**Figure 1:**
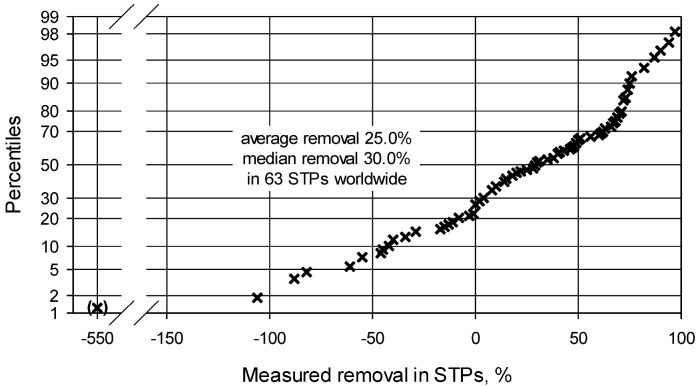
Distribution of 107 published degradation/removal rates of Trimethoprim (TMP) in 63 sewage treatment plants (STPs) worldwide.

For later PEC refinement, the recorded removal rates for full-scale working STPs were collated from 26 references [19,23,25,42,43,44,45,46,47,48,49,50,51,52,53,54,55,56,57,58,59,60,61,62,63,64] listed in Table A3 (with the exception of the above extreme value from [45]). The 107 remaining recorded removal rates, representing at least 63 STPs, ranged from −128% [48] up to >99% [63]. The average removal is 25.0% and the median removal 30.0% (Figure 1). These removal values are in agreement with the data by Fick *et al.* [60] who determined TMP to fall into an average removal range between 10% and 49% in their Swedish investigation in the year 2010. 

It is to be noted that all these empirically determined degradation rates depend on several circumstances, from time-corrected sampling of influents and effluents, to types and functional quality of the sewage works to the analytics themselves. While for the latter in most publications the analytical methods and recovery rates and ranges are described in detail, exact measured values are presented all the same, mostly without explicitly pointing out the uncertainty contained. This was recently shown by a group from Cleveland, Ohio sewage treatment works [77] who used two different contract labs to evaluate both intralaboratory and interlaboratory variability. They found discrepancies in TMP quantification of 40% in the same influent and of 168%–180% for the same effluents. This finding calls for caution in regarding all the measured concentrations of TMP (and other substances) as representing a true value; they could in fact be lower or higher.

##### 2.2.2.3. Environmental Fate of TMP (Table A4) [13,15,16,19,21,36,37,38,39,40,41,72,73,78]

Hydrolysis [65,66] and aquatic photodegradation in fresh and seawater [68,71] are not significant for TMP, except where both hydrogen peroxide and scavengers are present at the same time as UV irradiation [66,70,71,72]. Michael *et al.* [66] and Wu *et al.* [72] have recently confirmed that TMP degrades only slowly under natural solar illumination, approximately 10% in 500 min in demineralized water [66], respectively up to ~2% in 72 h in natural water [72]. However, it degrades much faster by hydrolysis in the aluminum-foil-wrapped dark control (up to ~15% in 72 h at *p*H 4 and 7), due to the temperature increase in the dark control [72]. While dissolved organic matter, which can act as a scavenger, is common in natural waters, peroxides may be less so; moreover, in most instances the superficial temperature will not rise massively, due to water movement. Hence, only slow photodegradation is predicted for TMP in temperate zones and it is not expected to play a major role in the environmental fate of TMP.

Total environmental half-lives (t½) of TMP have been derived for some compartments. In an experimental microcosm, Lam *et al.* [65] analytically determined a t½ of 5.7 ± 0.1 days; this short time may reflect the earlier findings of Bundschuh *et al.* [30] in their miniature fallen-leaf/natural-water system. Extrapolated, *i.e.*, estimated environmental half-lives for TMP are available for freshwater with >42 days [67] and 20–100 days [73]. Boxall *et al.* [67] also estimated a freshwater sediment t½ of >60–100 days, while Hektoen *et al.* [74] predicted a marine sediment t½ of 75–100 days. Once more there seems to be a wide range of half-lives for TMP, from the measured 5.7 ± 0.1 days [65] up to an estimated 100 days. This may again reflect nitrifying *vs.* non-nitrifying conditions, but mainly it does attest to a high uncertainty. 

However, the half-lives are important as the EU Technical Guidance Document for Risk Assessment (TGD) [79] classifies substances for persistence in function of their environmental half-life. Thereby, compounds are classified persistent (P) in freshwater if the aquatic half-life is >40 days and very persistent (vP) if it is >60 days; both P and vP in seawater if the marine half-life is >60 days; P if the freshwater sediment half-life is >120 days and vP if it is >180 days; P and vP if the freshwater or marine sediment half-life is >180 days [79]. Based on one experimental half-life of 5.7 days in a microcosm [65], TMP is not P, but it may well be P or even vP in freshwater if the extra­polated half-lives of >42 days [67] respectively 20–100 days [73] are correct.

##### 2.2.2.4. Bioaccumulation Data for TMP (Table A5) [9,28,47,67,80,81,82,83,84]

Data on bioaccumulation for TMP are scarce or indirect. The lipophilicity data for TMP range from a logKow of 0.64 to 1.15 [2,6,11,12], which argues against bioaccumulation. In an early experimental study, Bergsjø & Søgnen [9] exposed trout to a high TMP concentration of 75 mg/L in fresh and saltwater, but only for a short time of 84 h, which might not suffice for rigorous bioaccumulation assessment. They found a maximum bioconcentration factor (BCF; concentration in fish ÷ concentration in medium) of ~0.32 in marine fish liver and ~0.16 in freshwater fish liver, but from some of the graphs given the internal concentration seems to be still on the rise. However, Bergsjø *et al.* had dosed rainbow trout orally with radio-labeled TMP earlier [83] at a dose of roughly 0.02 mg TMP/g fish at 7 or 15 °C. Following the radio-label by autoradiography they noted a slow (maximum disintegrations per minute, DPM, around 48 h at 7 °C) to more rapid (max. DPM around 12–24 h at 15 °C) uptake, followed by a decrease that was rapid in muscle at 15 °C but slower in liver at 7 °C. Still, the maximum body concentration from a single dose reached at 48 h and declining thereafter does not speak for significant bioaccumulation. More recently, Fang *et al.* [81] dosed Japanese bass *(Lateolabrax japonicus)* once daily with 125 mg sulfamethazine and 25 mg TMP over five days. They derived the minimum holding period, unstated in the abstract but presumably until the analytes were below the limit of detection, from analysis in muscle, blood, liver and kidney as 26 days at 22 °C water temperature and 30 days at 16 °C. While no further information is given in the available abstract, a minimum 90% depuration time of 30 days at 16 °C does not seem inordinately long, suggesting reasonably rapid depuration and thereby relatively low bioaccumulation. Using multi-compartment, physico-chemical-activity-based modeling, Trapp *et al.* [28] showed that, contrary to expectation, TMP accumulates less in biota at *p*H 9 than at *p*H 6, due to increased relative partitioning to the sediment at *p*H 9 where TMP is mostly non-ionized. Conversely, according to Trapp and colleagues, at *p*H 6 TMP partitions more to biota than to sediment, but based on their data the worst-case water-biota BCF would still be <100 (approximate value from Figure 1 in Trapp *et al.*) [28]. This is indirectly supported by the reports of Ramirez *et al.* [82], who sampled common local fish from five wastewater-influenced streams in the eastern and southern USA as well as in one pristine control river, and Fick *et al.* [60], who did a comparable sampling in Swedish rivers and associated fish. Both groups never detected TMP in any of their fish samples. Fick and colleagues analyzed both surface water and biota samples at the same places; based on their range of surface water TMP concentrations, from 6.8 to 210 ng/L with no non-detects, and the fish concentration consistently below their LOQ of 0.1 µg/kg [60], the TMP BCF would be predicted to be <16 in the worst case. The regulatory limit for aquatic bioaccumulation is a BCF of 2000 for bioaccumulative (B) or of 5000 for very bioaccumulative (vB) according to the EU TGD [79]. Based on this mainly circumstantial evidence, TMP does not qualify as B. 

For uptake and bioaccumulation from spiked soil to plants over full growth duration for lettuce (103 days) and carrots (152 days), Boxall *et al.* [40] determined soil-based uptake factors of 0.06 for lettuce and 0.08 for carrots and soil-porewater-based uptake factors of 0.68 respectively 0.86 over the whole duration. Last, in a hydroponic exposure of two different sorts of cabbage plants, with 232.5 µg TMP/L in the nutrient solution over 51 days, Herklotz *et al.* [83] found a maximum wet-weight BCF of 0.3074. Even though for soil uptake in plants lower limits may apply for a B classification than for animals in water [85], with a BCF clearly <1 on chronic exposure there is no suspicion of bioaccumulation. Recently, Sabourin *et al.* [84] compared concentrations of pharmaceuticals and other substances in vegetables (sweet maize, carrot, tomato, potato) grown on soil fertilized with dried municipal sewage sludge or on non-amended control soil. Results for TMP are equivocal, as TMP was detected at comparable levels in tomatoes from one (0.432 ng TMP/g dry weight) of three amended soils and also from control soil (0.387 ng TMP/g dry weight), but was not detected in any other vegetable. The authors state that, by their own criterion that detections must be made in all three amended soils per vegetable, the results for all analytes including TMP are not significant. Hence, overall, there is no evidence for bioaccumulation of TMP. 

### 2.3. TMP Environmental Concentrations

#### 2.3.1. PECs and Use Data for Europe

The EMA 2006 Guideline for ERA of human pharmaceuticals [86] derives the initial, crude surface water PEC for APIs with a simple formula, multiplying the maximum daily dose with a default penetration factor in the population of 0.01 and dividing by a default 200 L sewage per person and day and a default surface water dilution factor of 10, without factoring in any human metabolism or STP removal. For TMP, with a daily dose of 400 mg [4], this results in a crude surface water PEC of 2 µg/L. However, this initial PEC may be refined through incorporating actual use, either through published epidemiological data resulting in a lower penetration factor or through actual use figures. 

IMS Health is a company that collates sales figures for APIs, hence total TMP sales (*i.e.*, pharmacies plus hospitals wherever available) were retrieved from the IMS Health database [87] for the years 1995–2003 for the following European countries: Austria, Belgium, France, Germany, Greece, Italy, The Netherlands, Portugal, Spain, Sweden, Switzerland and the United Kingdom, making up in 2003 a total of 370 million inhabitants [88]. Two results appear from this collation, first, the overall use declined from 55,578 kg in 1995 to 43,079 kg in 2003; such a decline was also noted by ter Laak *et al.* in a 2010 RIWA report on temporal and spatial trends of pharmaceuticals in the River Rhine [89] based on MECs in Dutch waters. Second, the average daily use per inhabitant for all these countries was 0.3955 mg TMP, with a range of 0.1937 mg for Greece to 0.5005 mg for the UK. For the last year in the series, the UK still has the highest *per capita* use per day of 0.5056 mg TMP.

Inserting the highest of the above daily use figures for the UK in 2003 into the PEC equation results in a first refined surface water PEC for the UK of 0.253 µg TMP/L. For the European 1995–2003 average use figure the first refined surface water PEC is 0.198 µg/L. 

This PEC may be further refined by excretion rate of the parent API including glucuronide or sulfate conjugates, which will be hydrolyzed back to the API in STPs [75]. Based on a maximum 20% of ingested TMP being Phase-1-metabolized [3], 80% excretion as the parent or its conjugates will be assumed as a worst case; 60% excretion will be assumed as a best case. This results in second refined surface water PECs of 0.202 µg/L for the UK in 2003, respectively of 0.119 µg/L for all European countries for 1995–2003.

A third PEC refinement may be made by incorporating STP removal of TMP. As derived above, based on a minimum of 107 measured removal rates, the average removal of TMP is 25.0% and the median (best case) removal is 30.0% (Figure 1). Using the lower, average removal for PEC refinement results in third refined surface water PEC of 0.152 µg/L for the UK in 2003, respectively of 0.089 µg/L for all European countries for 1995–2003. The serial PEC refinements are shown in Table 1.

**Table 1:** Surface Water predicted environmental concentrations (PECs) and their Refinement for TMP in Europe.

PEC stage	Surface water PEC, µg/L		Information used
	worst case	best case	
Initial crude	2.0		max daily dose, 400 mg [4], EMA ERA guideline [86]
First refinement	0.253	0.198	actual daily use per inhabitant, 0.5056 mg (maximum, UK) respectively 0.3955 mg (avg., Europe) (based on [87])
Second refinement	0.202	0.119	excretion rate, 80% respectively 60%
Third refinement	0.152	0.089	STP removal, 25.0% (avg.) respectively 30.0% (median)

Based on the available use, metabolism and STP removal data, a refined surface water PEC range of 0.089–0.152 µg/L seems realistic for Western Europe. This range can be compared with actual surface water MEC data.

#### 2.3.2. TMP MECs for Europe

TMP has been measured in European surface waters at least since the mid-1990s and today very many MECs can be located. In total, data representing at least 1899 single MECs have been collated for this ERA; ‘at least’, because often the number of single analyses is not given and in such cases just one value was assumed. Most of the publicly available MECs (at least 754) are from Germany [90,91,92,93,94,95,96,97,98,99,100,101,102,103]. Other MECs are from France [47,104], The Netherlands [105,106], Spain [5,48,107,108,109,110], Sweden [44,60,111], Switzerland [112,113,114,115,116], Croatia [49] and the United Kingdom [50,117,118,119,120]. Special thanks to F Bonvin, T Kohn, M Lehmann and M Schärer (see Acknowledgements) for supplying single MEC data that have only been published as overviews.

**Figure 2:**
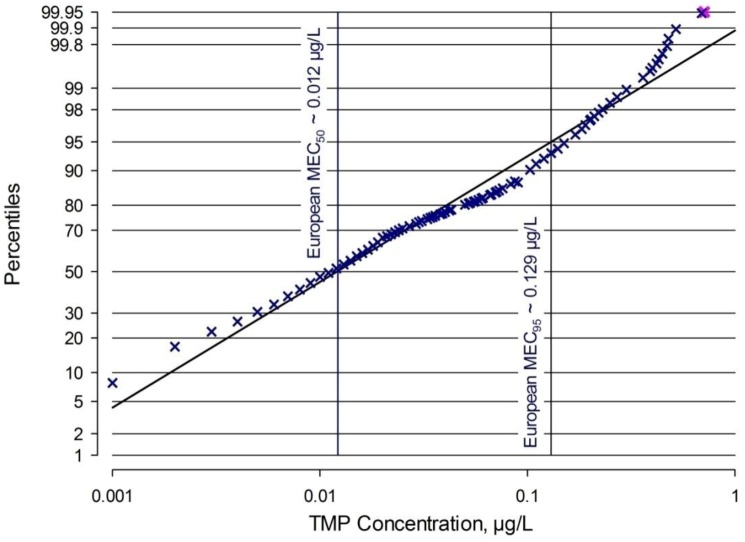
Compiled European surface water measured environmental concentrations (MECs) for TMP.

The values were collated into one single distribution as described by Straub [121,122] and Metcalfe *et al.* [123], detailed in the Experimental Section further below. Figure 2 is based on at least 1899 back-distributed single measurements that were percent-ranked. Datapoints (blue crosses) were inserted at those concentrations where at least one MEC is either explicitly reported or can be allocated with certainty. In view of many MECs being reported as below the limit of detection or quantitation, there are already a cumulative 8 percentiles of all MECs at 0.001 µg/L, corresponding to an estimated 150 MECs. The 50th and 95th percentiles (MEC_50_ respectively MEC_95_) are indicated in Figure 2 by drop lines; the MEC_50_ is ~0.012 µg/L, the MEC_95_ ~0.129 µg/L. For comparison, the highest single surface water MEC located in the literature, from the USA [124], is 0.710 µg/L (pink cross in Figure 2), very close to the highest European MEC of 0.690 µg/L [109].

It is recognized that this procedure does not deliver exact results but, on the other hand, it is the only possibility of compiling different MEC data into one single distribution and getting a consolidated overview comprising all data, instead of many smaller distributions presented in different formats. Moreover, the more data there are in this distribution, the better will it reflect the actual environmental distribution, in particular at the 95th percentile level, where indeed most references and their respective MEC values are fully integrated already.

#### 2.3.3 Comparison of TMP PECs and MECs for Europe

The MEC_95_ and MEC_50_ values lend themselves for comparison with the refined PEC (and alter also the PNEC) values. Recalling the refined PEC range of 0.089–0.152 µg/L, it would seem that the higher PEC is close to the MEC_95_ but that the best-case PEC is a factor of ~7.5 higher than the MEC_50_. Both PECs, however, could actually be too high, possibly for the following reasons.

- The PECs assume that the whole amount sold is also used and excreted. Patient noncompliance seems to be relatively common, however [125,126,127]. Particularly with antibiotics, some patients stop taking the medicines when they start to feel better, without finishing the whole treatment course. As long as these discarded APIs are not drained into the wastewater, this will reduce the surface water PEC.
- The PECs assume that the average and median removal rates in STPs derived here are representative for the whole of Europe. Possibly more STPs have well nitrifying AS that results in higher removal and thereby in a lower surface water PEC.
- The PECs assume a TGD [79] default surface water dilution factor of 10. If the average dilution factor in Europe is higher this would result in a lower PEC.
- The PECs do not factor in environmental degradation beyond the STPs. However, TMP can be degraded by both aerobic and anaerobic biological mechanisms [30,65] and to some degree by physico-chemical transformation, also in surface waters [70,72]. Both would reduce the PEC.

### 2.4. TMP Environmental Effects and Predicted No Effect Concentrations

#### 2.4.1. Micro-organism/STP Inhibition

For STPs to perform their intended function, the AS micro-organisms must not be affected by the micropollutants in the influent. Hence an appraisal of bacterial toxicity of TMP is necessary, the basic data are collated in Table A6 [15,18,21,29,30,64,128,129,130,131,132,133,134,135].

TMP is not highly toxic to AS bacteria in standard aerobic and anaerobic tests [18,21,128], with EC50 values ranging from 17.8 to >100 mg/L. Also, in Lumistox tests with the light-emitting marine bacterium *Vibrio fischeri* TMP is not highly toxic, however, toxicity increases with prolonged exposure [129,130,131]. On even longer exposure of 14 days, TMP completely inhibited human nanobacteria at 3.9 mg/L [132]; this would be expected as the human therapeutical serum concentration is in the range of 1.5–9 mg/L [3]. In a closed bottle ready biodegradation test, no inhibition was noted in the standard toxicity control at 3.25 mg/L TMP-naphthoate, while a significant reduction of colony-forming was noted at 4.6 µg/L TMP-naphthoate [29]. This possible discrepancy is not discussed in the paper, however, the toxicity control measures overall inhibition while the colony-forming units relate to cultivable bacterial species. Hence the observations by Alexy *et al.* [29] may signify that TMP exerts adverse effects only on certain bacterial species, which may be masked or compensated by the remaining, non-affected species in AS. This interpretation may be supported by the findings of a statistical EC10 in AS of 0.435 mg/L (in contrast to the NOEC observed at 100 mg/L in the same GLP test) [129], by NOECs to soil bacteria of 0.02 mg/L [133] and to nitrifying bacteria at 0.05 mg/L in one test [57], while another nitrification inhibition test under GLP showed no effect at 96 mg/L [134]. Moreover, when tested in combination with four other antibiotics (sulfamethoxazole, erythromycin, roxithromycin, clarithromycin; all at the same concentration), TMP reduced the growth of fungi on fallen alder leaves in natural water at 40 µg/L, while at 0.4 µg/L no inhibition was noted [30]. 

Altogether, the above findings are rather difficult to interpret. It seems that TMP can inhibit certain microbial species at concentrations of 4.6 µg/L (LOEC) [29] while other bacteria are not adversely affected at concentrations over 100 mg/L [21]. In an STP, the latter may take over some of the ecological functions of the affected species, as suggested by the AS respiration inhibition and nitrification inhibitions tests (both relying on overall functional endpoints), and thereby compensate functionally for the inhibited micro-organisms. This is supported by the observation that biodegradation (not only of TMP itself but in general) and nitrification in working STPs is not significantly inhibited by the influent concentrations of TMP and many other substances, as shown by overall functional parameters. Therefore, TMP may cause inhibition of specific bacteria and potentially shifts in species compositions at concentrations between 4.6 and 0.4 µg/L, but at current uses there is no evidence of adverse effects on the functions of STPs. This may also be related to a certain tolerance (or resistance) of STP bacterial communities toward many different micropollutants including TMP. 

Similarly, Liu *et al.* [135] found in an experiment with spiked natural soil that TMP decreases the total soil respiration in comparison with a blank control during the first 4 days of exposure from 20 mg TMP/kg soil (dry weight), whereas from day 5 to the end of the assay at day 21 no inhibition was noted, but either no change or increased respiration at all concentrations up to the highest of 300 mg TMP/kg soil (dry weight). This was interpreted as an initial overall inhibition followed by an adaptation of the collective of aerobic micro-organisms. In this work, Liu *et al.* [135] note a soil dissipation half-time (DT50) of 2–5 days for TMP and in a later publication the same group [15] gives a DT50 in aerobic soil of 4 days, which suggests that after about half of the spiked TMP is removed (by biodegradation or bound residue formation) the bacterial community adapts to the substance. In view of the very general endpoint of total respiration a persistent inhibition of certain species is still conceivable, but the ongoing dissipation of TMP in the soil through mainly biodegradation [15] suggests that in such a case at least the biodegradation functionality can be compensated by the remaining bacteria.

#### 2.4.2. Surface Water Ecotoxicity

For the appraisal of surface water ecotoxicity there are two extensive datasets for TMP, one acute (Table A7) and one chronic (Table A8). The acute dataset fully rests on published and some older Roche-internal tests (which are already used for the Roche safety data sheets) while for the chronic dataset some new tests performed specifically for this ERA are reported for the first time.

##### 2.4.2.1. Acute Ecotoxicity of TMP (Table A7) [9,18,124,129,130,136,137,138,139,140,141,142,143,144,145,146,147,148,149,150]

Acute data exist for cyanobacteria, algae, hydrozoans, rotifers, crustaceans, molluscs, flowering plants and fish. The acute EC50 or LC50 data range from 5.1 mg/L for a marine alga (where TMP would be mostly non-dissociated in view of the basic *p*H of seawater) [137] to 296 mg/L for daphnids [149] for those tests where a concise value is given (*i.e.*, not a ‘>highest tested concentration’). For fish in particular, all highest tested concentrations did not result in an LC50, which would be expected in view of the fact that fish are not intended to be target organisms for antibiotics. The one apparent exception to this is an LC50 value of 3 mg/L cited by Kolpin *et al.* [124], which proves to be a miscitation: The original paper by Bergsjø *et al.* [80], which is actually referred to by Kolpin *et al.* [124], gives a single oral dose of approximately 0.02 mg radio-labelled TMP per gram of fish, but not a concentration. Moreover, none of the fish used is reported by Bergsjø *et al.* [80] to have died of TMP. Hence, this erroneous citation is not used for toxicity assessment.

Antibiotics are used to inhibit bacterial infections, which is why Holten Lützhøft *et al.* [138] noted that ‘to perform a proper environmental risk assessment of antibacterial agents, it would be necessary to include a cyanobacteria as test organism in the test battery’; this request has been adopted in the EMA guideline for ERA of human APIs [86]. But at least in the case of TMP the cyanobacterian species tested are neither the most sensitive nor is the range of cyanobacterian EC50s limited to low concentrations; on the contrary, the EC50s range from 11 to >200 mg/L [136,138]. This may suggest that for some reason TMP is not as highly toxic to cyanobacteria than to human nanobacteria [132]; possibly, photosynthetic cyanobacteria are not as dependent on their own *de novo* folate biosynthesis as human pathogenic bacteria. By extension, the comparatively high threshold for ecotoxicological effects over the broad array of groups and species tested confirms the statement by Blaise *et al.* [129] that TMP is relatively nontoxic.

##### 2.4.2.2. Chronic Ecotoxicity of TMP (Table A8) [136,137,139,140,141,142,143,144,145,146,151,152,153,154]

The new chronic tests under GLP quality assurance commissioned with the aquatic flowering plant *Lemna minor* [143] and the zebrafish *Danio rerio* [153] bring the total number of systematic groups tested chronically to 8 (including the three standard groups algae, daphnids and fish) and the number of species to 17. Once more, the marine alga that was already the most sensitive on an acute scale has the lowest EC50 [137], which (again as expected) suggests that TMP would be more toxic while mostly non-dissociated in view of the basic *p*H of seawater. Also on a chronic level the cyanobacterians have a wide range of NOECs, from 3.1 to ≥200 mg/L [136]. 

A FETAX larval test with the toad *Xenopus laevis* is included among the chronic data despite the short duration, as this test takes place during a very sensitive phase of development and has therefore been accepted as chronic by the recent EU Technical Guidance Document for Deriving Environmental Quality Standards (EQS) in the scope of the EU Water Framework Directive [155]. Together with the new zebrafish early life stage NOEC at the highest tested concentration of 100 mg/L [153], the fish and amphibian data once more suggest that vertebrates are not particularly sensitive to TMP and that generally speaking, also on a chronic level TMP is relatively nontoxic [129] as well.

### 2.5. TMP Predicted No Effect Concentrations

#### 2.5.1. Deterministic TMP PNEC Derivation

This copious compilation of acute and chronic ecotoxicity data allows for both a solid deterministic and a well-founded probabilistic PNEC or HC5 (hazardous concentration for 5% of species tested) according to the requirements of the TGD [79]. Where more than one result was available for the same species, the geometrical average was calculated and this will be used for PNEC derivation, in line with the EU EQS derivation guidance [155]. According to the TGD [79], when at least three endpoints are available for a minimum dataset of algae, daphnids and fish, the acute deterministic PNEC is the lowest EC50 or LC50 divided by an assessment factor (AF) of 1000; the chronic deterministic PNEC is the lowest NOEC or EC10 divided by an AF of 10.

The lowest chronic value retrieved, a NOEC of ≥1 mg/L (the highest tested concentration) for the duckweed *Lemna gibba* [142] will not be used for deterministic or probabilistic PNEC derivation, however, because (a) basing a PNEC on a lower bound of a NOEC generates a high uncertainty in general, in particular (b) because from a ‘≥’ value no unambiguous deterministic or probabilistic PNEC can be derived, but again only a ‘≥’ value, and (c) because the closely related *Lemna minor* showed a clear NOEC of 53.5 mg/L in a GLP test [143] with measured exposure concentrations, about 50 times higher than the disputed value. 

In addition, other, very low, highest tested concentrations published without any biological effects noted whatsoever, like the above value in Brain *et al.* 2004 [142], were not used. This concerns the daphnid NOEC of 10 µg/L (highest tested concentration) published by Flaherty & Dodson 2005 [152], which included the endpoints survival, adult and neonate morphology, ephippium production, fecundity and offspring sex ratio. However, it was based on a duration of only 6 days, whereas the OECD guideline stipulates 21 days, and was therefore not used for derivation of PNECs. Also, in a recent test with the marine rotifer *Brachionus koreanus*, Rhee *et al.* [145] tested nominal concentrations of 10 and 100 µg/L TMP for 10 days and noted ‘gradual’ or ‘slight growth retardation’ [145] (pp. 109 and 116, respectively) at 100 µg/L. However, while a slight retardation in growth may be seen from their graph on p 115, Rhee and colleagues do not comment on the fact that TMP-exposed *Brachionus* seem to fully compensate their delayed reproduction by the end of the test on day 10, when the error bars of controls and the two tested concentrations overlap. As the test runs over ten days, as there is no significant adverse effect at the end of the test and as the authors did not test sufficiently high concentrations to unambiguously demonstrate such an effect, this endpoint will not be used here. 

Last, the biomarker data for the zebra mussel *Dreissena polymorpha* published by Binelli *et al.* [156] are not used for PNEC derivation, either, as the acute-based NOEC is based on ambiguous inhibition or mortality endpoints. The same holds for the biomarker endpoints in the rotifer paper by Rhee and colleagues [145]. For the time being there is no regulatory guidance on extrapolation from biomarker responses to organism- or population-relevant endpoints that may be used within the scope of an ERA. Rejecting them for the PNEC derivation is in line with the EU EQS guidance document [155] which states that ‘data from studies describing endpoints that do not include direct measurements of survival, development or reproduction but, rather, describe e.g., behavioral effects, anatomical differences between control and treatment groups, *effects at the tissue or sub-cellular level, such as changes in enzyme induction or gene expression* … generally … are unsuitable as the basis for EQS derivation’. 

Based on these provisions the deterministic acute-based aquatic PNEC for TMP is 5.1 µg/L, derived from the marine algal EC50 of 5.1 mg/L (*Phaeodactylum tricornutum*) [137], applying an AF of 1000. Also the deterministic chronic aquatic PNEC of 240 µg/L relies on the same algal species with a NOEC of 2.4 mg/L [137] and an AF of 10. The chronic-based PNEC is considered more relevant in view of reflecting long-term, continuous exposure. However, the fact that the most sensitive organism for both the acute and chronic endpoints is a marine alga, suggests that the high *p*H of seawater renders TMP more toxic due to a higher non-dissociated fraction, beside the algae-typical phenomenon of ion trapping [157]. 

#### 2.5.2 Probabilistic PNEC Derivations

##### 2.5.2.1. TGD Probabilistic PNEC

**Figure 3:**
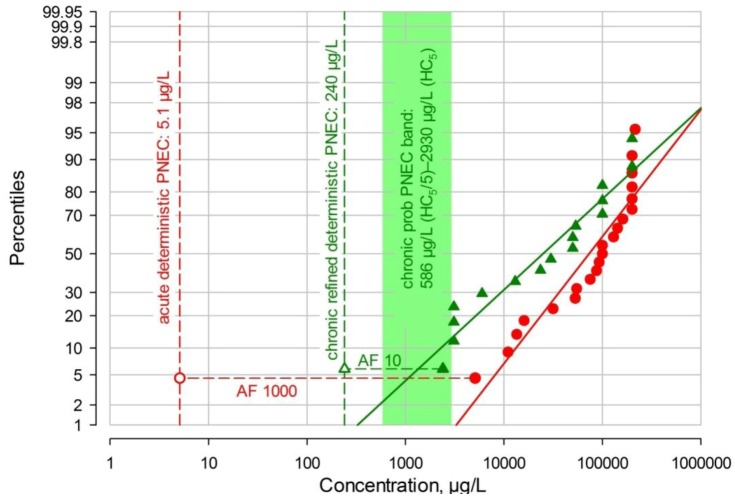
Acute and chronic ecotoxicity data, deterministic and probabilistic predicted no effect concentrations (PNECs) for TMP.

The first probabilistic PNEC was derived as described in the EU TGD [79] by calculating the HC5 or 5th percentile of the chronic NOECs distribution and dividing this figure by an additional AF between 1 and 5. While there is some information given on the choice of this additional AF, no unequivocal, hard criteria exist. Hence for this ERA, a range for the chronic probabilistic PNEC will be given, from HC5/5 to HC5. The HC5 calculated by Excel is 2.93 mg/L, therefore the probabilistic PNEC range is 586–2,930 µg/L, with an average of 1,758 µg/L. The derivation of the PNECs is shown graphically in Figure 3.

In Figure 3, the acute aquatic EC50/LC50 values (red dots) and chronic aquatic NOEC values (filled dark green triangles) for TMP are shown, both percent-ranked and plotted on a log-probabilistic scale, with deterministic PNECs (open symbols; AF 1000 for acute data, AF 10 for chronic NOECs) and the light green TGD-calculated probabilistic PNEC band ranging from HC5÷5 (586 µg/L) to the HC5 (2,930 µg/L).

##### 2.5.2.2. Webfram Probabilistic HC5

**Figure 4:**
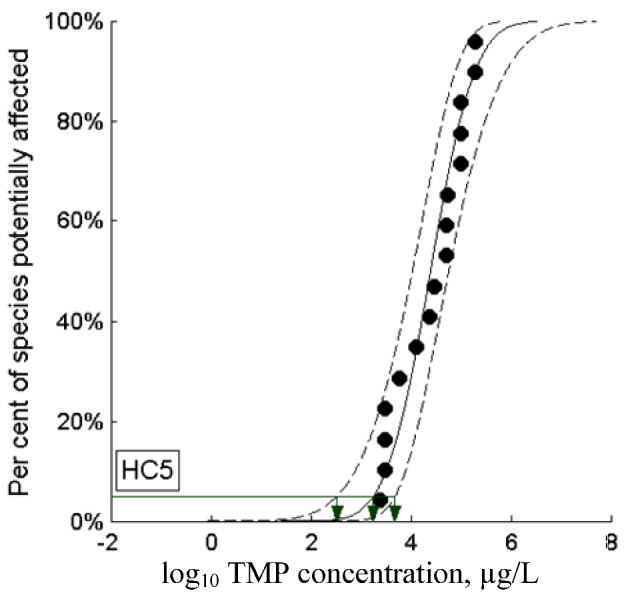
Webfram: chronic aquatic NOEC values and HC5. Chronic aquatic NOEC values (black dots) for TMP, percent-ranked and plotted by Webfram on a log-probabilistic scale; the 95% confidence interval is given as dashed lines. The Webfram-calculated probabilistic HC5 is 1,778 µg/L (middle green arrow) and the 95% confidence interval for the HC5 lies between 334 and 4,832 µg/L (left and right green arrows).

In addition to the TGD probabilistic PNEC, the chronic NOECs were entered into the Webfram application (http://www.webfram.com) [158], which calculates a probabilistic HC5 based on a Bayesian algorithm [159]. Moreover, Webfram also computes goodness-of-fit values according to Kolmogorov-Smirnov, Cramer-Von Mises and Anderson-Darling algorithms; for all three tests the goodness-of-fit of the chronic TMP NOECs is accepted at a *p* value of 0.01. The probabilistic HC5 as determined by Webfram is 1,778 µg/L, with a 95% confidence interval between 334 and 4,832 µg/L (Figure 4). This HC5 compares nicely with the average of the EU probabilistic PNEC range, 1,758 µg/L. 

### 2.6. Aquatic Environmental Risk Assessment for Human-Use TMP in Europe

#### 2.6.1. TMP Risk Characterization Ratios

With sufficient exposure and effects information, both transformed into PECs or MECs and PNECs, the formal ERA for the surface waters in Europe can now be addressed. The various PECs and compiled MECs are compared with the PNECs in Table 2.

All risk characterization ratios without exception are <1, which means no significant risk overall. In particular, all risk characterization ratios that use any chronic-based, deterministic or probabilistic PNEC, which is taken to better reflect the permanent exposure to APIs, range from <0.01 to <0.00001. This firmly corroborates the first conclusion of no significant risk from TMP in surface waters in Europe and beyond.

**Table 2:** TMP risk assessment for European surface waters: PECs, MECs, PNECs and PEC/PNEC and MEC/PNEC risk characterization ratios respectively margins of safety.

Environmental concentrations (PECs and MECs)		Predicted no-effect concentrations (PNECs)		Risk ratio (PEC/PNEC or MEC/PNEC)	Margin of safety (inverse of risk ratio)
**Derivation**	**value, µg/L**	**Derivation**	**value, µg/L**		
EMA crude PEC	2.0	acute-det	5.1	0.392	2.55
	2.0	chronic-det	240	0.00833	120
	2.0	chronic-pr	586–2930	0.00341–0.000683	293–1465
	2.0	Webfram pr HC5	1778	0.00112	889
Third refined PEC (incl. actual use, excretion rate, STP removal) [this work]	0.152–0.089	acute-det	5.1	0.0299–0.0175	33.6–57.3
	0.152–0.089	chronic-det	240	0.000633–0.000371	1579–2697
	0.152–0.089	chronic-pr	586–2930	0.000259–0.0000304	3855–32921
	0.152–0.089	Webfram pr HC5	1778	0.0000855–0.0000500	11697–19978
European MEC95 [this work, Figure 2]	0.129	acute-det	5.1	0.0253	39.5
	0.129	chronic-det	240	0.000538	1860
	0.129	chronic-pr	586–2930	0.000220–0.0000440	4543–22713
	0.129	Webfram pr HC5	1778	0.0000726	13783
European MEC50 [this work, Figure 2]	0.012	acute-det	5.1	0.00235	425
	0.012	chronic-det	240	0.00005	20000
	0.012	chronic-pr	586–2930	0.0000205–0.0000041	48833–244167
	0.012	Webfram pr HC5	1778	0.00000675	148167
Maximum European MEC [109]	0.690	acute-det	5.1	0.135	7.39
	0.690	chronic-det	240	0.00286	348
	0.690	chronic-pr	586–2930	0.00118–0.000235	849–4246
	0.690	Webfram HC5	1778	0.000388	2577
Maximum MEC located worldwide, USA [124]	0.710	acute-det	5.1	0.139	7.18
	0.710	chronic-det	240	0.00296	338
	0.710	chronic-pr	586–2930	0.00121–0.000242	825–4127
	0.710	Webfram pr HC5	1778	0.000399	2504

#### 2.6.2. TMP Risk Graph

**Figure 5:**
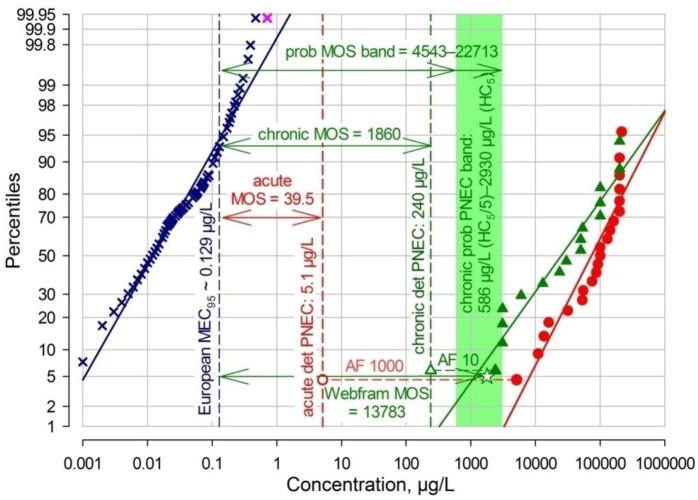
TMP risk graph for European surface waters.

The whole information for this ERA including the margins of safety determined here can also be illustrated in one single risk graph for TMP (Figure 5).

In the risk graph (Figure 5) the whole exposure and effects information for TMP is brought together. Acute aquatic EC50/LC50 data are shown as red dots, with the derivation of the deterministic PNEC of 5.1 µg/L (hollow red circle) by application of an assessment factor (AF) of 1000. Chronic aquatic NOEC values are shown as filled dark green triangles, with the derivation of the deterministic chronic PNEC of 240 µg/L (hollow green triangle) by application of an AF of 10. Further, the bright green probabilistic TGD chronic PNEC band ranging from 586 to 2,930 µg/L is depicted as well as the Webfram-calculated HC5 of 1,778 µg/L (green star in the band). European MECs are shown by dark blue crosses, with the European MEC_95_ at 0.129 µg/L; in addition, the highest MEC from the USA of 0.710 µg/L is shown as a pink cross. For illustration, selected margins of safety (MOS) are shown by horizontal arrows from the MEC_95_ to the corresponding PNECs. 

Beyond the MOSs between the MEC95 for Europe and selected PNECs, the risk graph also shows very clearly that, at least within the confines of the 1st and 99.95th percentiles, the MEC regression lines and the chronic NOECs regression line do not overlap. This illustrates graphically that there is no perceivable risk. In view of the fact that TMP use has been declining in Europe over the past 10–15 years, this conclusion is further strengthened.

#### 2.6.3. Limitations of the Present TMP ERA

##### 2.6.3.1. Mixture Assessment

Synergistic or cocktail effects arising from the exposure to many micropollutants, comprising not only APIs but quite a diverse group of substances, are not included in this ERA. However, the data collated and presented here can serve for developing the TMP ERA further to include at least some other APIs, mainly sulfamethoxazole or other sulfonamides, with which TMP is often combined. But while some aspects of mixtures ERA are reasonably well understood [160], it is not easy to do a combined ERA for a few substances and it becomes practically impossible to do it for a large number. Hence, the present TMP ERA does not address mixture toxicity.

##### 2.6.3.2. Human Plus Veterinary Use of TMP

The PECs on which this ERA relies only refer to human use of TMP. But TMP is also used on a large scale for veterinary purposes, again mostly in combination with sulfonamides. While total European quantitative data are not readily available, there are both veterinary and human use data published for Denmark over the past 15 years (DANMAP) [161]. Denmark is a European country with intense agricultural production, both of farm animals like pigs, cows or poultry as well as of fish in freshwater and marine aquaculture. Hence, extrapolating from the Danish data to the European level is judged to add a worst-case exposure from animal use of TMP. DANMAP 2012 data show that the total veterinary usage of TMP plus sulfonamides has been rising in the decade from 2001 to 2010, with a maximum of 14,950 kg in 2009 and the 2010 figure at 13,900 kg. Assuming also a 5:1 ratio of veterinary sulfonamides to TMP (as with human sulfamethoxazole and TMP in Bactrim) would translate to an annual veterinay use of 2,333 kg TMP for Denmark. Specifically for aquaculture, 3,060 kg antimicrobials were used in 2010, of which 66% or 2,020 kg sulfonamides plus TMP, which again corresponds to 337 kg TMP for direct aquatic usage and 1,996 kg TMP (2,333 minus 337) for mammals and poultry. On the human-use side, in 2010, 417 kg TMP and derivatives were used beside 252 kg of sulfonamides plus TMP, which latter amount translates to 42 kg TMP, hence a total of 459 kg TMP from human use. Assuming that the farm animal use will not get directly into surface waters and therefore adding only the aquaculture TMP, which is used directly in water, to the total human use, results in a supplement of 337 kg to the 459 kg, or 73% more. Hence, as a very crude worst-case extrapolation, 173% of the human-use PECs will be used as an overall surface water PEC from human plus veterinary use for ERA. Multiplying the various PECs in Table 2 (above) with a factor of 1.73 will increase the PEC/PNEC ratios, but even for the rather unrealistic EMA crude PEC of 2.0 µg/L, now increased to 3.46 µg/L, the acute-based risk characterization ratio is still <1; it is still lower by dimensions for the MECs (which at least for Denmark include that part of veterinary TMP that ends up in surface waters) as well as for chronic PNECs. Hence, even including a reasonable worst-case contribution from veterinary use to aquatic TMP PECs will not lead to a significant surface water risk.

##### 2.6.3.3. Antibiotic Resistance

Another topic that is far beyond the scope of this ERA is antibiotic resistance development or maintenance due to the presence of antibiotics like TMP in STPs, surface waters or other environmental compartments [162,163]. While multi-antibiotic resistance has been shown for certain environmental compartments, notably sewage treatment, surface waters and soils [164,165], it is difficult to causally relate solely the presence of antibiotics (as opposed to the input of resistant bacteria from human patients or livestock) to the development or maintenance of such resistance. Indeed, some researchers found no maintenance, but on the contrary loss, of resistance in a laboratory sewage treatment plant despite the continued presence of antibiotics [166]. Moreover, so far there is no accepted regulatory methodology to assess this question. Hence, the question of potential resistance must remain for other investigations.

##### 2.6.3.4. Further Environmental Compartments

According to the TGD ERA methodology [79], substances may be transferred from wastewater to the soil by way of land-spreading of surplus sewage sludge and from surface water to sediment by partitioning or to groundwater by infiltration. For all of these pathways there are insufficient data for a serious assessment of TMP, both on the environmental fate, distribution, partitioning or MEC side and in particular on the effects side in the receiving compartments. In view of this situation, no attempt will be made to characterize risk for these compartments by discussing the meager data or by read-across. 

## 3. Experimental

### 3.1. Literature Search

Environmentally relevant peer-reviewed and non-reviewed (‘grey’) literature for TMP was searched for using dedicated search engines on the internet (ACS SciFinder, Google Scholar, chemical data collections like OECD Chemicals Portal http://www.echemportal.org/ or the European Union Chemical Substances Information System http://esis.jrc.ec.europa.eu/ as well as safety data sheet search engines such as https://www.eusdb.de), beside company-internal substance documentation and archives. The information was sighted, ordered and collated. Reference lists in the retrieved documents often allowed to supplement the literature dataset with further, mostly older publications and also online sources for MECs. 

### 3.2. Collation of STP Removal Rates and Surface Water MECs

All retrieved published STP removal rates, *viz. *effluent concentration as a percentage of influent concentration, worldwide were entered into a spreadsheet with removal rates ranging from −550% (the highest negative removal reported, which eventually was not used in the analysis, see argument on page 118) to 100% removal, with a value of 1 per documented removal rate into one column per each reference. All rates were horizontally added to a total per removal rate in per cent. Then, these values were multiplied by 100 and divided by the known total number of removal rates plus 1, in a percent-ranking procedure. Then, a plot was drawn using SigmaPlot 12 software (Scistat, Inc., San Jose, CA, USA) with the percentiles on a probabilistic ordinate and the removal rates on a linear abscissa. The average and median removal rates were determined by excel spreadsheet functions.

All reported, discrete, single European surface water MECs were entered into one column per reference into a spreadsheet with a 1-ng/L-gradation ranging from ≤1 ng/L up to 1,000 ng/L. Then, the remaining (non-specified) MEC data were back-distributed per publication into the same column, based on total number of analyses, number below LOQ, between LOQ and median, between median and 90th percentile and between 90th percentile and the maximum value, to an average expected fraction or number of detections per ng/L-gradation for these ranges. For instance, if the LOQ in a particular publication was 5 ng/L and there were 7 MECs <LOQ, 7 was divided by 5 and the resulting fraction of 1.4 was entered into all 5 gradations from ≤1 to ≤5. Similarly, the number of MECs given between LOQ and the median, or between the median and the 75th or 90th percentile if indicated, were back-distributed. Then, both the precisely known numbers and the expected, back-distributed fractions of detections were horizontally added per ng/L-gradation, multiplied by 100 and divided by the known total number of analyses plus 1, in a percent-ranking procedure. This procedure resulted in a theoretical 690 values computed, 0.690 µg/L being the highest published surface water MEC in Europe, from a series that sampled only 100 m downstream of sewage works effluents in Madrid Region [109]. Out of these 690 values, however, only those values were kept for plotting and graphical regression where at least one actual analytical detection was certain. The plot was drawn using SigmaPlot software with a probabilistic ordinate and a logarithmic abscissa. The associated regression line then allows the graphical estimation of the overall 50th and 95th percentile MEC values (MEC_50_ respectively MEC_95_) based on at least 1899 single European MECs (Figure 2).

### 3.3. Identification of Ecotoxicity Data Gaps and Additional Ecotoxicity Studies

Based on the chronic aquatic ecotoxicity dataset retrieved and critically analyzed, it was found that chronic fish studies were totally lacking and that a chronic study with the angiosperm *Lemna gibba* [142] was not adequate for risk assessment as the NOEC found was the highest tested concentration. To fill these data gaps, two additional chronic ecotoxicity studies with the duckweed *Lemna minor* following OECD test guideline 221 [143] and the zebrafish *Danio rerio* following OECD TG 210 were commissioned at reliable contract labs. Moreover, an activated sludge respiration inhibition test according to OECD TG 209 [128] and a dedicated activated sludge nitrification inhibition test following ISO TG 9509 [134] were also made. All newly commissioned tests were performed under GLP quality assurance, in the cases of the duckweed growth inhibition and the fish early life stage tests also with full analytical determination of the exposure concentrations by HPLC and statistical determinations of EC_10_ and EC_50_s as applicable, beside the NOECs. All additional tests were financed by Roche.

### 3.4. Risk Assessment Methodology

Deterministic and probabilistic ERA methods were applied, following the EU TGD [79] for both acute- and chronic-based deterministic PNEC derivation as well as for TGD probabilistic PNEC band calculation. Additionally, the Webfram online tool (http://www.webfram.com) [158] was used for deriving a second probabilistic PNEC or HC_5_ based on a Bayesian algorithm.

## 4. Conclusions

An extended ERA was developed for the aquatic compartment in Europe for the old antibiotic TMP from human use. This ERA relies on both crude and refined surface water PECs for TMP, the latter integrating actual use figures, human metabolism and documented STP removal rates; these PECs range from the crude EMA PEC of 2 µg/L to the third refined PEC of 0.089 µg/L. The PECs are complemented by a veritable host of at least 1899 single MECs from European countries that were compiled into one distribution, allowing the approximation of median (0.012 µg/L) and 95th percentile (0.129 µg/L) values for surface water concentrations, with the European maximum at 0.690 µg/L. 

On the environmental effects side, existing and newly developed ecotoxicity data were used to derive deterministic acute and chronic PNECs of 5.1 respectively 240 µg/L. The 16 chronic data from 8 different systematic groups were also used to derive a probabilistic PNEC range of 586–2,930 µg/L (EU TGD) or a probabilistic HC5 (PNEC) value of 1,778 µg/L (95% CI: 334–4,832 µg/L; Webfram). All acute (EC50/LC50) and chronic (NOEC/EC10) ecotoxicity data for cyanobacteria, green algae, marine algae, angiosperms, hydrozoans, rotifers, crustaceans, fish and amphibians are above 1 mg/L, supporting low ecotoxicity for TMP. 

All PEC/PNEC or MEC/PNEC risk characterization ratios are <1, all of the chronic-based risk ratios are <0.01 to <<0.01, showing no indication of risk due to the presence of TMP in surface waters. 

Moreover, while the available data suggest that TMP is persistent in surface waters, there is no evidence that TMP bioaccumulates and there are no experimental ecotoxicity data that suggest inordinately high toxicity; hence TMP is not a PBT substance, either.

Based on this extended ERA, no significant risk is seen for TMP from human use in the aquatic compartment in Europe.

Insufficient environmental fate and effects data were available for a reasonably well founded ERA for the compartments sediment and soil, but evidence is given that these two compartments in all probability are not central for TMP from human use. Also, there is a plausibility presentation that the additional veterinary use of TMP does not lead to significantly increased surface water levels and thus not to significant increased risk. The issues of mixture toxicity and antibiotic resistance could not be addressed based on available data and risk assessment procedures.

## Appendix

**Table A1:** Physico-Chemical Data for TMP.

Property	Method	Value	Unit	Reference
CAS number		738-70-5		SDS Roche [2]
Molecular mass		290.32	g/mol	SDS Roche [2]
Melting point	experimental	199–203	°C	SDS Roche [2]
Vapour pressure	experimental	9.88 × 10^–9^ = 1.32 × 10^–6^	mm Hg Pa	Gros *et al.* 2006 [5]
Water solubility	experimental	400	mg/L, 25 °C	PhysProp online [6]
	experimental	400	mg/L	Chen *et al.* 2002 [7]
	experimental	401	mg/L	Ran *et al.* 2002 [8]
	experimental	300	mg/L	SDS Roche [2]
	experimental, freshwater & marine	~75 (both)	mg/L	Bergsjø & Søgnen 1980 [9]
Dissociation constant	experimental	7.6	base pKa	Bergsjø & Søgnen 1980 [9]
	experimental	7.2; 6.6	base pKa	Clarke’s online [3]
	experimental	6.6	base pKa	Roche SDS [2]
	experimental	6.76 ± 0.12; 3.23 ± 0.30	base pKa1 base pKa2	Qiang & Adams 2004 [10]
Octanol/water partition coefficient	experimental	0.64	logKow	Roche SDS [2]
	experimental	0.74, *p*H 7.4	logD	Zhu *et al.* 2002 [11]
	experimental	0.91	logKow	PhysProp online [6]
	experimental	1.115	logKow	Zhao *et al.* 2002 [12]
Adsorption to organic carbon, Koc	experimental	1680–3990	L/kg	Boxall *et al.* 2005 [13]
Koc, digested sludge	experimental	724 (logKoc = 2.86)	L/kg	Barron *et al.* 2009 [14]
Koc, soil	experimental	224 (logKoc = 2.35)	L/kg	Barron *et al.* 2009 [14]
Koc, soil	experimental, soil *p*H 4.9	719	L/kg	Liu *et al.* 2010 [15]
Koc, soil	experimental	4600	L/kg	Lin & Gan 2011 [16]
Koc	QSAR estimate	2692	L/kg	Franco & Trapp 2010 [17]
Sorption (Kd) to activated sludge (AS)	experimental	76	L/kg	Halling-Sørensen *et al.* 2000 [18]
Kd, AS	experimental	208 ± 49	L/kg	Göbel *et al.* 2005 [19]
Kd, AS	experimental	~200–300	L/kg	McArdell *et al.* 2005 [20]
Kd, AS	experimental inherent bodegradability test	~1500 (3 h), ~966 (28 d)	L/kg	Gartiser *et al.* 2007 [21]
Kd, AS	experimental	330 ± 25	L/kg	Abegglen *et al.* 2009 [22]
Kd, digested sludge	experimental	68	L/kg	Barron *et al.* 2009 [14]
Kd, primary sludge	experimental	427 ± 238	L/kg	Radjenovic *et al.* 2009 [23]
Kd, AS	experimental	253 ± 37	L/kg	Radjenovic *et al.* 2009 [23]
Kd, membrane bioreactor	experimental 2 MBRs	225 ± 87; 320 ± 117	L/kg	Radjenovic *et al.* 2009 [23]
Kd, AS	experimental	68	L/kg	Power *et al.* 2009 [24]
Sorption to AS	experimental	‘negligible’		Batt *et al.* 2006 [25]
Sorption WWTP	experimental	‘negligible’		Göbel *et al.* 2007 [26]
Kd, AS	experimental	7.4; but strong adsorption in one soil	L/kg	Lin & Gan 2011 [16]
Kd, soil	experimental	26	L/kg	Power *et al.* 2009 [24]
Kd, soil	experimental, soil *p*H 4.9	9.7	L/kg	Liu *et al.* 2010 [15]
Sorption to sludges	experimental	ND in primary, secondary and digested sludge as well as in compost		Martín *et al.* 2012 [27]

**Table A2:** Biodegradability and elimination of TMP.

Test Type	Inoculum	Endpoint	TMP Conc, mg/L	Duration	Degradation	Reference
Ready biodegradability OECD301F		BOD/ThOD	19.4		0%	Halling-Sørensen *et al.* 2000 [18]
Ready biodegradability OECD 301D		BOD/ThOD	3.25 (TMP-naphtoate)	28 day	4%	Alexy *et al.* 2004 [29]
Ready biodegradability OECD 301D, toxicity control/ cometabolic degradation		BOD/ThOD	3.25 (TMP-naphtoate) plus sodium acetate	28 day	27%	Alexy *et al.* 2004 [29]
Degradation in a water/leaf system	fallen leaves, natural water	substance loss	0.04	168 h	~80%	Bundschuh *et al.* 2009 [30]
Inherent respirometric test (Roche-internal)	mixed industrial-municipal AS	BOD/ThOD	200	5 day	0%	Gröner 1981 [31]
Inherent biodegradability		t½ primary degradation	0.5	22–41 day		Halling-Sørensen *et al.* 2000 [18]
Inherent biodegradability (combined Zahn-Wellens/ CO2 evolution test)		DOC, BCO2	100 mg TOC/l	28 day	negative (toxic to sludge)	Gartiser *et al.* 2007 [21]
Inherent biodegradability	nitrifying AS with long SRT (49 d)	substance loss	0.25	96 h	~70%	Batt *et al.* 2006 [25]
Inherent biodegradability	nitrifying AS with long SRT (49 d)	degradation half-life	0.25	~67 h		Batt *et al.* 2006 [25]
Inherent biodegradability OECD 303A	AS	substance loss	0.03 radio-labelled	21 day	<1%	Junker *et al.* 2006 [32]
Inherent biodegradability	AS with 220 d SRT	substance loss	0.001		74%	Yu *et al.* 2009 [33]
Inherent bio-degradability, small membrane bioreactor	AS	primary degradation constant k_biol_			0.22 ± 0.022 l × g_ss_^–1^d^–1^	Abegglen *et al.* 2009 [22]
(Inherent) Biodegradability	primary sewage	primary degradation	0.02	54 day	~40%, slow	Pérez *et al.* 2005 [34]
(Inherent) Biodegradability	AS	primary degradation	0.02	54 day	NS/slight increase	Pérez *et al.* 2005 [34]
(Inherent) Biodegradability	nitrifying sludge	primary degradation	0.02	3 day	100%, rapid	Pérez *et al.* 2005 [34]
Elimination	primary wastewater treatment				–13% to 31%	Göbel *et al.* 2007 [26]
Elimination	conventional AS with 10–25 d SRT				–40 ± 20% to 20 ± 11%	Göbel *et al.* 2007 [26]
Elimination	AS with 60–80 d SRT				87%–90%	Göbel *et al.* 2007 [26]
Elimination	fixed-bed reactor				12 ± 11% to 17 ± 11%	Göbel *et al.* 2007 [26]
Elimination	pilot membrane bioreactors in a WWTP	substance loss	50 µg/L	SRT 15 day & HRT 9 h; SRT 30 day & HRT 13 h	86% SRT 15; 94% SRT 30	Schröder *et al.* [35]
Elimination	sand filter				15%–74%	Göbel *et al.* 2007 [26]
Elimination	sand filter				60%	Göbel *et al.* 2005 [19]

**Table A3:** Removal of TMP during sewage treatment.

Sewage treatment plants (STP)	Type	Measurement		Removal	Reference
STPs Germany	AS	substance loss, two analytical methods		18 ± 14%, 29 ± 17%	Ternes *et al.* 1999 [42]
STPs Europe (n = 7)	AS	substance loss		0%, 4×<10%, 30%, 40%	Paxéus 2004 [43]
STPs Switzerland (n = 2)	AS	substance loss		74%	Göbel *et al.* 2005 [19]
STP Sweden	AS	substance loss		49%	Bendz *et al.* 2005 [44]
STP Sweden (n = 2)	AS	substance loss		–550% (!) to 68%	Lindberg *et al.* 2005 [45]
STP Sweden	AS	substance loss		−45%, −1%, 40%	Lindberg *et al.* 2006 [46]
STP France	AS	substance loss		51%	Paffoni *et al.* 2006 [47]
STP Spain	AS	substance loss		−128% to 71%	Gros *et al.* 2007 [48]
STPs Croatia (n = 2)	AS	substance loss		−15%, 49%	Senta *et al.* 2008 [49]
STPs Wales (n = 2)	AS	substance loss		47%, 70%	Kasprzyk-Hordern *et al.* 2009 [50]
STP Spain (n = 2)	AS	substance loss		40.4 ± 25.4%	Radjenovic *et al.* 2009 [23]
STPs Spain (n = 2)	membrane bioreactor	substance loss		66.7 ± 20.6% 47.5 ± 22.5%	Radjenovic *et al.* 2009 [23]
STPs Canada (n = 2)	AS	substance loss		14 ± 2%, NS 38 ± 4%	Segura *et al.* 2006 [51]
STP USA	AS	substance loss		~50%	Batt *et al.* 2006 [25]
STP USA	AS	substance loss		69%	Brown *et al.* 2006 [52]
STP USA (n = 4)	various	substance loss		50%, 61%, 66%, 67%, 69%, 83%	Karthikeyan & Meyer 2006 [53]
STP USA	nitrifying AS	substance loss	influent >0.01 µg/L (LOD), effluent <LOD	not quantified	Levine *et al.* 2006 [54]
STPs USA (n = 4)	AS	substance loss		70%, 76%, 82%, 97%	Batt *et al.* 2007 [55]
STP Australia	AS	substance loss		85%	Watkinson *et al.* 2007 [56]
STPs Japan (n = 4)	different secondary treatments	substance loss		−88%, −82%, −46%, 35%, 63%, 73%, 74%	Ghosh *et al.* 2009 [57]
STP China (n = 4)	different primary and secondary treatments	substance loss		−42%, −17%, −11%, 42%	Gulkowska *et al.* 2008 [58]
STP Norway (n = 1)	AS	substance loss		–60% to 28%, values only from graph	Plósz *et al.* 2010 [59]
STP Sweden (n = 4)	AS	substance loss		4%, 13%, 63%, 76%; average 39%	Fick *et al.* 2011 [60]
STPs Ireland (n = 3)	AS	substance loss		0–94.6%	Lacey *et al.* 2012 [61]
STPs Hong Kong/China (n = 7)	different secondary treatments	substance loss		43% overall removal	Leung *et al.* 2012 [62]
STP Taiwan (n = 1)	primary, seconday & tertiary	substance loss		>99%	Lin *et al.* 2012 [63]
STPs Spain (n = 2)	AS	substance loss		8%, 29%	Verlicchi *et al.* 2012 [64]

**Table A4:** Environmental Fate of TMP.

Endpoint	Medium	Measurement	Conditions	Duration	Result	Reference
Hydrolysis					stable	Lam *et al.* 2004 [65]
Hydrolysis					stable	Michael *et al.* 2012 [66]
Aquatic photodegradation					not readily photodegradable	Boxall *et al.* 2002 [67]
Aquatic photodegradation				42 day	no photodegradation	Boxall *et al.* 2004 [68]
Aquatic photodegradation	seawater, natural sunlight			21 day	stable	Lunestad *et al.* 1995 [69]
Aquatic photodegradation	Hg-Nd lamp, H_2_O_2_, tap water			10 min; 20 min	>90%; >99%	Türk 2007 [70]
Aquatic photodegradation					<10% UV only; up to 92% with UV, H_2_O_2 _and scavengers	Rosario-Ortiz *et al.* 2010 [71]
Aquatic photodegradation	natural sunlight	substance loss	2 mg/L, *p*H 4,7&9	72 h	slight degradation during daytime only, up to ~2% at 72 h	Wu *et al.* 2011 [72]
Aquatic photodegradation	natural sunlight	substance loss	2 mg/L, aluminium-wrapped dark control, *p*H 4,7&9	72 h	increased degradation up to ~15% (*p*H 4 & 7) correlating with temperature	Wu et al 2011 [72]
Aquatic photodegradation	natural sunlight	substance loss	10 mg/L demineralised water	500 min	increased with Fenton reagent, decreased in simulated and natural wastewater	Michael *et al.* 2012 [66]
Ozonation					rapid destruction	Türk 2007 [70]
Environmental half-life	freshwater microcosm	t½ measured		5.7 ± 0.1 day		Lam *et al.* 2004 [65]
Environmental half-life	freshwater	t½ estimate		>42 day		Boxall *et al.* 2002 [67]
Environmental half-life	freshwater	t½ estimate		20–100 day		Zuccato *et al.* 2001 [73]
Environmentalhalf-life	marine sediment	t½ estimate		<60–100 day		Boxall *et al.* 2002 [67]
Environmental half-life	marine sediment	t½ estimate		75–100 ayd		Hektoen *et al.* 1995 [74]
Elimination	freshwater sediment	primary degradation		14 h	15%	Löffler & Ternes 2003 [36]
Riverbank filtration		substance loss			>75% removal	Schmidt *et al.* 2006 [37]
Anaerobic biodegradability ISO11734		methane production			NS	Gartiser *et al.* 2007 [21]
Anaerobic degradability	surplus sludge digestion	primary degradation			>99%	Göbel *et al.* 2005 [19]
Anaerobic biodegradability VDI 4630	manure & anaerobic bacteria	primary degradation (LC/MS)	2.8 mg/kg; 14 mg/kg	34 day	98.9% day 8; 99.9% day 9	Mohring *et al.* 2009 [38]
Anaerobic degradation	pig slurry				rapid degradation	Grote *et al.* 2004 [39]
Sewater degradation	seawater	DT50	0.001	>100 day		Benotti & Brownawell 2009 [41]
Soil degradation	soil	DT50		110 day		Boxall *et al.* 2005 [13]
Soil dissipation	soil	DT50, DT90		<103 day, >152 day		Boxall *et al.* 2006 [40]
Soil dissipation	aerobic, non-sterile	DT50	10 mg/kg	4 day		Liu *et al.* 2010 [15]
Soil dissipation	aerobic, sterile	DT50	10 mg/kg	64 day		Liu *et al.* 2010 [15]
Soil dissipation	anaerobic, non-sterile	DT50	10 mg/kg	11 day		Liu *et al.* 2010 [15]
Soil dissipation	anaerobic, sterile	DT50	10 mg/kg	79 day		Liu *et al.* 2010 [15]
Soil degradation	aerobic soil	percentage of loss attributed to biodegradation	10 mg/kg	49 day	~28%	Liu *et al.* 2010 [15]
Soil degradation	anaerobic soil	percentage of loss attributed to biodegradation	10 mg/kg	49 day	~56%	Liu *et al.* 2010 [15]
Soil degradation	aerobic soil		40 µg/kg dry weight	t½ = 26.1 day	*note:* no significant anaerobic degradation, no degradation in sterilised soil, nor in another soil	Lin & Gan 2011 [16]
Removal during soil passage	aerobic turfgrass soil, sampled at ~90 cm depth	substance loss during leaching			91%–98%	Bondarenko *et al.* 2012 [78]

**Table A5:** Bioaccumulation data for TMP.

Bioaccumulation	Organism	Organ	Dosage	Duration	Result	Reference
Bioconcentration freshwater	fish, trout	autoradiographs	single oral dose	up to 144 h	maximum concentrations given as DPMs only reached at 12–24 h (15 °C) respectively 48 h (7 °C), then rapid decline in both cases	Bergsjø *et al.* 1979 [80]
Bioconcentration freshwater	fish, trout	liver, muscle, plasma		84 h	~0.16; ~0.04; ~0.01	Bergsjø & Søgnen 1980 [9]
Bioconcentration marine	fish, trout	liver, muscle, plasma,		84 h	~0.2–0.32; ~0.08–0.12; ~0.03–0.07	Bergsjø & Søgnen 1980 [9]
Bioconcentration aquatic	physico-chemical activity-modelled				higher predicted TMP concentration in biota at *p*H 6 than at *p*H 9 due to increase in sediment concentration at *p*H 9	Trapp *et al.* 2010 [28]
Depuration marine	fish, Japanese seabass	muscle, blood,liver, kidney	5 oral doses, one per day, of 125 mg sulfamethazine and 25 mg TMP	minimum holding period after last dose 26 days at 22 °C, 30 days at 16 °C		Fang *et al.* 2003 [81]
Biomonitoring freshwater USA: 5 wastewater-influenced rivers, 1 pristine control	fish (various local species)	muscle, liver	not measured	permanent (wild fish)	ND (<2.2); ND (<8.0) LODs in ng/g	Ramirez *et al.* 2009 [82]
Biomonitoring freshwater Sweden: 4 wastewater-influenced rivers, 2 pristine controls	fish, perch	muscle		permanent (wild fish)	ND (<0.1 ng/g LOQ)	Fick *et al.* 2011 [60]
Bioaccumulation plants	lettuce and carrots *(Daucus carota)*	lettuce leaf, carrot root	1 mg/kg soil dry weight	103 days lettuce, 152 days carrots	soil-based uptake factor lettuce 0.06, carrot 0.08; porewater-based uptake factor lettuce 0.68, carrot 0.86	Boxall *et al.* 2006 [40]
Bioaccumulation plants	2 cabbage cultivars	leaf/stem root	232.5 µg/L hydroponic nutrient solution	51 days	bioaccumulation factor 0.0383–0.3074 (wet weight), 0.0451–7.037 (dry weight)	Herklotz *et al.* 2010 [83]
Bioaccumulation plants	sweet maize, carrot, tomato, potato		field fertilised with dehydrated sewage sludge (biosolids)		equivocal/ NS	Sabourin *et al.* 2012 [84]

HRT = Hydraulic retention time; LOD = limit of detection; ND = not detected; NS = not significant; SRT = sludge retention time.

**Table A6:** Micro-organism and activated-sludge toxicity data.

Organism/Sludge	Systematic Group	Endpoint	Duration	Value, mg/L	Reference
AS, OECD209		EC50		17.8	Halling-Sørensen *et al.* 2000 [18]
AS, OECD209		EC50; EC20	3 h	>200; 19	Oggier/BMG 2011, GLP [128]
Anaerobic sludge inhibition ISO13641		EC50	7 days	>100	Gartiser *et al.* 2007 [21]
*Vibrio fischeri*	bacteria, marine	IC50	15 min	183.3	Blaise *et al.* 2006 [129]
*Vibrio fischeri* ISO 11348–3	bacteria, marine	IC50	15 min	176.7	Kim *et al.* 2007 [130]
*Vibrio fischeri*	bacteria, marine	IC50	30 min	23.3	Isidori *et al.* 2005 [131]
Human nanobacteria	bacteria	MIC	14 days	3.9	Ciftcioglu *et al.* 2002 [132]
AS, OECD209	bacteria	NOEC; EC10	3 h	100; 0.435	Oggier/BMG 2011, GLP [128]
AS in Closed Bottle ready biodegradation test OECD301D	bacteria	NOEC toxicity control		3.25 mg/L TMP-naphthoate	Alexy *et al.* 2004 [29]
AS in Closed Bottle ready biodegradation test OECD301D	bacteria	LOEC colony-forming units		4.6 µg/L TMP-naphthoate	Alexy *et al.* 2004 [29]
*Pantoea agglomerans*	soil bacterium	NOEC		0.02	Tappe *et al.* 2006 [133]
Nitrification inhibition test	nitrifying bacteria	NOEC		0.05	Ghosh *et al.* 2009 [57]
Nitrification inhibition test	nitrifying bacteria	NOEC; EC10		96; >96	Oggier/BMG 2011, GLP [134]
Fungal growth on fallen leaves	fungi	LOEC; NOEC	TMP together with 4 other antibiotics, all at same conc	40 µg/L; 0.4 µg/L	Bundschuh *et al.* 2009 [30]
Natural soil respiration	all aerobic soil microorganisms	EC10 (0–4 days)		20 mg/kg soil (dry weight)	Liu *et al.* 2009 [135]
Natural soil respiration	all aerobic soil microorganisms	after 4 days consistent increase in respiration *vs.* controls in all concentrations up to the highest of 300 mg/kg soil		300 mg/kg soil (dry weight)	Liu *et al.* 2009 [135]
Natural soil	bacteria (colony-forming units)	NOEC/LOEC		10 mg/kg	Liu *et al.* 2010 [15]

**Table A7:** Acute ecotoxicity data for TMP.

Organism	Systematic Group	Endpoint	Duration	Value, mg/L	Reference
*Anabaena cylindrica*	Cyanobacteria	EC50	6 days	***>200***	Ando *et al.* 2007 [136]
*Anabaena flos-aquae*	Cyanobacteria	EC50	6 days	***>200***	Ando *et al.* 2007 [136]
*Anabaena variabilis*	Cyanobacteria	EC50	6 days	***11***	Ando *et al.* 2007 [136]
*Microcystis aeruginosa*	Cyanobacteria	EC50	7 days	112	Holten Lützhøft *et al.* 1999 [138]
*M. aeruginosa*	Cyanobacteria	EC50	6 days	150	Ando *et al.* 2007 [136]
*M. aeruginosa*	Cyanobacteria	EC50		***129.6***	***geometrical average***
*Microcystis wesenbergii*	Cyanobacteria	EC50	6 days	***>200***	Ando *et al.* 2007 [136]
*Nostoc* sp. PCC7120	Cyanobacteria	EC50	6 days	***53***	Ando *et al.* 2007 [136]
*Synechococcus leopoldensis*	Cyanobacteria	EC50	6 days	***>200***	Ando *et al.* 2007 [136]
*Synechococcus* sp. PCC7002	Cyanobacteria	EC50	6 days	***>200***	Ando *et al.* 2007 [136]
*Rhodomonas salina* ISO 8692	Algae, marine	EC50	72 h	***16***	Holten Lützhøft *et al.* 1999 [138]
*Phaeodactylum tricornutum*	Algae, marine	EC50	72 h	***5.1***	Claessens *et al.* 2009 [137]
*Pseudokirchneriella subcapitata (=Selenastrum capricornutum)*	Algae	EC50	72 h	40	Yang *et al.* 2008 [139]
*P. subcapitata*	Algae	EC50	72 h	80.3	Eguchi *et al.* 2004 [140]
*P. subcapitata*	Algae	EC50	72 h	96.7	Blaise *et al.* 2006 [129]
*P. subcapitata* OECD 201	Algae	ErC50	72 h	98	Bogers 1996a GLP [141]
*P. subcapitata* ISO 8692	Algae	EC50	72 h	110	Halling-Sørensen *et al.* 2000 [18]
*P. subcapitata*	Algae	EC50	72 h	130	Holten Lützhøft *et al.* 1999 [138]
*P. subcapitata*	Algae	EC50	72 h	***87.1***	***geometrical average***
*Lemna gibba*	Angiospermae	EC50	7 days	>1 HTC	Brain *et al.* 2004 [142]
*Lemna minor* OECD 221	Angiospermae	ErC50	7 days	***215***	this work, GLP, Oggier 2011 [143]
*Hydra attenuata*	Cnidaria	EC50	96 h	>85.3	Blaise *et al.* 2006 [129]
*H. attenuata*	Cnidaria	EC50	96 h	>100	Quinn *et al.* 2008a [144]
*H. attenuata*	Cnidaria	EC50	96 h	***>92.4***	***geometrical average***
*Brachionus koreanus*	Rotatoria (brackish)	EC50	24 h	198.5	Rhee *et al.* 2012 [145]
*Daphnia magna*	Crustacea	EC50	48 h	92	Park & Choi 2008 [146]
*D. magna* OECD 202	Crustacea	EC50	48 h	>100 HTC	Bogers 1996b GLP [147]
*D. magna* US EPA 600/4_90/027	Crustacea	EC50	48 h	123	Halling-Sørensen *et al.* 2000 [18]
*D. magna*	Crustacea	EC50	48 h	149	De Liguoro *et al.* 2009 [148]
*D. magna*	Crustacea	EC50	48 h	167.4	Kim *et al.* 2007 [130]
*D. magna*	Crustacea	EC50	96 h	296	Iannacone & Alvariño 2009 [149]
*D. magna*	Crustacea	EC50	48 h	***142.4***	***geometrical average***
*Moina macrocopa*	Crustacea	EC50	48 h	***54.8***	Choi *et al.* 2008 [150]
*Thamnocephalus platyurus*	Crustacea	EC50	24 h	***161.2***	Blaise *et al.* 2006 [129]
*Crassostrea gigas*	Mollusca, marine	EC50 embryolarval	24 h	***~31.6***√(10×100)	Claessens *et al.* 2009 [137]
*Danio rerio* OECD 203	Fish	NOEC	72 h	100	Halling-Sørensen *et al.* 2000 [18]
*D. rerio*	Fish	NOEC	96 h	100	Blaise *et al.* 2006 [129]
*D. rerio*	Fish	LC50	96 h	***>100***	***geometrical average***
*Oryzias latipes*	Fish	LC50	96 h	***>100***	Kim *et al.* 2007 [130]
*Oncorhynchus mykiss*	Fish	LC50	84 h	**>75 **HTC	Bergsjø & Søgnen 1980 [9]
*O. mykiss*	Fish	LC50	96 h	(3) *note:* miscitation, not a concentration but a dose	miscited in Kolpin *et al.* [124]

*Note:* In case of several values for the same species, the geometrical average was calculated [155]. Values in ***bold italics*** are the values used for PNEC derivation while the single value in brackets was not used for the PNEC, see text. HTC = Highest tested concentration.

**Table A8:** Chronic Ecotoxicity Data for TMP.

Organism	Systematic Group	Endpoint	Duration	Value, mg/L	Reference
*Anabaena cylindrica*	Cyanobacteria	NOEC	6 days	***≥200***	Ando *et al.* 2007 [136]
*Anabaena flos-aquae*	Cyanobacteria	NOEC	6 days	***≥200***	Ando *et al.* 2007 [136]
*Anabaena variabilis*	Cyanobacteria	NOEC	6 days	***3.1***	Ando *et al.* 2007 [136]
*Microcystis aeruginosa*	Cyanobacteria	NOEC	6 days	***100***	Ando *et al.* 2007 [136]
*Microcystis wesenbergii*	Cyanobacteria	NOEC	6 days	***3.1***	Ando *et al.* 2007 [136]
*Nostoc* sp. PCC7120	Cyanobacteria	NOEC	6 days	***3.1***	Ando *et al.* 2007 [136]
*Synechococcus leopoldensis*	Cyanobacteria	NOEC	6 days	***13***	Ando *et al.* 2007 [136]
*Synechococcus* sp. PCC7002	Cyanobacteria	NOEC	6 days	***50***	Ando *et al.* 2007 [136]
*Phaeodactylum tricornutum*	Diatom Algae, marine	NOEC	72 h	***2.4***	Claessens *et al.* 2009 [137]
*Pseudokirchneriella subcapitata (=Selenastrum capricornutum)*	Green Algae	NOEC	72 h	16	Yang *et al.* 2008 [139]
*P. subcapitata*	Green Algae	NOEC	72 h	25.5	Eguchi *et al.* 2004 [140]
*P. subcapitata*	Green Algae	NOEC	72 h	32	Bogers/NOTOX 1996a GLP [141]
*P. subcapitata*	Green Algae	NOEC	72 h	***23.5***	*geometrical average*
*Lemna gibba*	Angiospermae	NOEC	7 days	(>1 HTC) not used*	Brain *et al.* 2004 [142]
*Lemna minor*	Angiospermae	NOEC	7 days	***53.5***	this work, GLP Oggier 2001 [143]
*Hydra attenuata*	Cnidaria	NOEC	96 h	>100	Quinn *et al.* 2008a [144]
*H. attenuata*	Cnidaria	NOEC	96 h	25	Quinn *et al.* 2008b [151]
*H. attenuata*	Cnidaria	NOEC	96 h	***>50***	***geometrical average***
*Brachionus koreanus*	Rotatoria (brackish)	NOEC/LOEC	10 days	(0.01/0.1) not used*	Rhee *et al.* 2012 [145]
*Daphnia magna*	Crustacea	NOEC	21 days	***6***	Park & Choi 2008 [146]
*Daphnia magna*	Crustacea	NOEC	6 days	(0.01) not used*	Flaherty & Dodson 2005 [152]
*Moina macrocopa*	Crustacea	NOEC	21 days	***≥30*** HTC	Park & Choi 2008 [146]
*Danio rerio*	Fish	NOEC	35 days	***100***HTC	this work, GLP, Gilberg & Hamberger 2011 [153]
*Xenopus laevis*	Amphibia	EC10	96 h	***≥100***	Richards & Cole 2006 [154]

Note: In case of several values for the same species, the geometrical average was calculated. Values in ***bold italics*** are the values used for PNEC derivation. HTC = Highest tested concentration. Endpoints/ values in brackets were not used. * = See text.
